# Emergent patterns of interaction with dynamic objects

**DOI:** 10.1371/journal.pone.0331844

**Published:** 2025-09-18

**Authors:** Buse Aktas¸, Paris Myers, Emily Salem, Roberta L. Klatzky, Robert D. Howe

**Affiliations:** 1 Robotic Composites and Compositions Group, Max Planck Institute for Intelligent Systems, Stuttgart, Germany; 2 John A. Paulson School of Engineering and Applied Sciences, Harvard University, Boston, Massachusetts, United States of America; 3 MIT Media Lab, Massachusetts Institute of Technology, Cambridge, Massachusetts, United States of America; 4 Department of Psychology and Human-Computer Interaction Institute, Carnegie Mellon University, Pittsburgh, Pennsylvania, United States of America; University of Marburg: Philipps-Universitat Marburg, GERMANY

## Abstract

Perception by touch is fundamentally linked to the motor system. A hallmark of this linkage takes the form of stereotyped haptic “exploratory procedures” [1], movement patterns that emerge when people set a perceptual goal such as judging the roughness of a textured surface. This paper expands the study of touch-directed movements by asking what patterns emerge when people encounter and interact with novel objects without explicitly specified goals. Participants were invited to freely interact with an art installation containing novel objects with distinct design features, intended to vary familiarity, structural affordance, and aesthetic response. Objects’ affordances were additionally varied over time by utilizing jamming, a physical mechanism that induces changes in stiffness and plasticity. From video recordings, four categories of spontaneous “interactive procedures” differentiated by underlying goals were reliably identified: passive observational, active perceptual, constructive, and hedonic. Perceptual actions were most frequent, indicating an overriding goal of acquiring information about physical properties. The prevalence of other interactive procedures varied across objects, demonstrating the influence of perceptual affordances and prior knowledge. Changes in state further moderated interactions, such that interactions were longer in the stiff/jammed state, and the occurrence of a state change during an interactive procedure lengthened its duration. These findings extend our understanding of haptic exploration beyond explicitly goal-directed contexts, revealing how spontaneous responses in complex and dynamic environments  are linked to perceptual outcomes and prior knowledge.

## Introduction

In everyday experience, we interact with the world around us through touch to obtain information about objects and to perform tasks with objects. Studies have shown what kinds of actions (“exploratory procedures”) we perform when we have specific perceptual goals [1], and why these actions are optimal for the designated purpose [2–5]. Other psychological studies have quantified how vision and touch work together during haptic interactions [6,7] and how the form and material of an object change interaction and perception [8,9]. Most such prior studies, however, focus on human behavior in controlled experimental conditions, utilizing constrained object sets and setting specific perceptual goals; for example, to compare objects on the basis of surface roughness. In natural contexts, in contrast, people are often observed to explore objects without an external directive, but instead for internally generated goals, and objects may be subject to change during interaction. While it has been shown that certain object properties increase this need for touch and that there are variations across individuals [10,11], the nature of the specific haptic interactions has not been systematically described. There is a lack of prior work studying what **interactive procedures** emerge when people are engaging in unstructured contexts with objects subject to dynamic change. The purpose of the present study was to characterize the nature of these open-ended haptic interactions and to identify factors that guide and modulate exploratory behaviors.

Two pathways that might guide interactions with free-standing objects are considered. “Top-down” guided actions stem from expectations based on prior experience and context, such as “an object in this category produces sound when shaken” or “this object seems to be inflated and might yield to squishing” [12–14]. “Bottom-up” actions, on the other hand, are induced by the features of the objects themselves, or object *affordances* [15]. Affordances not only present physical possibilities, but also invite behavior and shape the interaction [16–18]. Visual affordances arise, for example, as shape contours indicate potential for grasping or matching surface geometries invite conjoining objects. Haptic affordances are also available from initial contact, as when an object slips or yields, conveying the potential for lifting or deforming. Bottom-up influences extend beyond physical interactions, as evidenced by findings that visual complexity and artistic abstraction affect engagement vs. distancing from artifacts [19,20].

Additionally, object affordances can change over the course of interaction. Physical features of objects are subject to change in state, for example, by softening, swelling, or modifying color. Changes can be induced by the interaction itself, such as when ice cubes melt in our hands or clay dries and hardens as we try to shape it. Such dynamic changes in interactions are also becoming more salient as engineers and scientists continue to develop active functional robotic materials and structures that can be incorporated into human-system interactions [21–26]. There is a need to expand our current understanding of human-object interactions, which has primarily been focused on objects with static properties, to incorporate the effects of active matter. The contribution of this study lies not only in systematically describing and characterizing emergent interactive procedures in unstructured contexts, but also in examining the effects of changes in object affordances throughout the course of the interaction.

**Fig 1:**
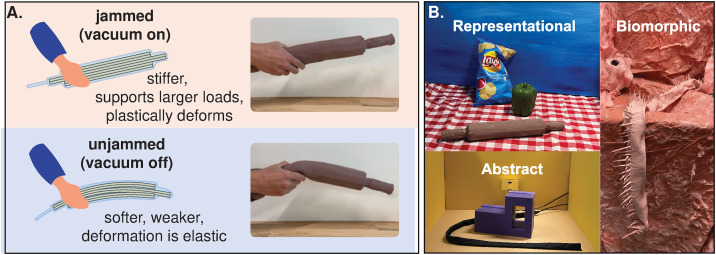
Jamming-based objects with dynamic mechanical properties were created and incorporated into a multi-station interactive haptic installation for participants to freely interact with. **(A)** Vacuum-based jamming structures, which are made of an airtight elastic membrane filled with constituents of different geometries (fibers, granules, sheets), can transition between a high-stiffness and plastically deformable state and a low-stiffness and elastic state. **(B)** The setup included three separate stations. The representational station had a potato chip bag, a pepper, and a rolling pin. The abstract station had one purple geometric form and a black strip. The biomorphic station had a pink intestine-like tubular structure and a fuzzy drape spanning multiple surfaces. All the listed objects were jammed and unjammed in varying time periods.

One unstructured context that motivates spontaneous haptic exploration is art exhibitions, as evidenced by the necessity of signs that say “do not touch.” To experimentally observe such emergent behavior, the present study created an interactive haptic art installation with novel objects. To enhance the impression of an art exhibit, the forms of the objects were inspired from a range of artistic styles and movements. The installation comprised three separate stations, termed representational, biomorphic and abstract. To varying degrees across stations, expectations and affordances were induced by using objects with prior familiarity (e.g., bag of chips), structural regularity (polygonal grooves and holes), and unformed suggestive forms (e.g., intestine-like structure with biomorphic features). In addition to identifying categories of interactive procedures, our goal was to examine how people’s exploratory activities at each station are modulated by their static and dynamic features, indicating underlying goals of freely generated haptic interaction (See Fig 1).

To observe spontaneous interactive responses to state change, the haptic installation incorporated a state-changing potential to all objects, based on the mechanism of jamming. A jamming structure typically consists of a collection of elements with low effective stiffness. When a pressure gradient, such as a vacuum, is applied, the kinematic and frictional coupling between elements increases, resulting in dramatically altered mechanical properties such as stiffness, damping, and plasticity [27–29]. Taking advantage of the versatility of morphologies and mechanical properties possible with jamming, engineers have built jamming-based devices such as conformable grippers, orthotic devices, surgical manipulators, and haptic interfaces [26,30–32]. Employing jamming in the multi-station installation introduced the possibility to periodically stiffen and soften all interaction objects and observe the effects of these changes in state.

In order to describe the different interactive procedures that emerge throughout participants’ engagement with the dynamic and novel objects, a novel classification system was developed. It was anticipated that existing classifications for haptic exploration would not be sufficient. Previously identified exploratory procedures directed at perceptual features [2,4] were expected to emerge, but unlikely to cover the range of interactions. Unlike many of the typical experiments on haptic exploratory procedures where participants are blindfolded, vision is present in these interactions. Therefore, it was expected that behavioral signals that evidence visual engagement with objects would emerge [33–35]. In addition, since the interaction was self-directed and unconstrained, manipulatory actions [36] as well as hedonic and playful actions were expected. As reported below, the framework for interactive behaviors led to reliable classification and allowed insights into how object properties and dynamic state changes influence ongoing haptic interaction.

## Materials and methods

Three different table-top booth-like stations were created, each with a variety of jamming-based objects. Each booth was about 75 cm wide, 50 cm deep, and 50 cm high. The **representational station** contained everyday objects: a green bell pepper, a rolling pin and a bag of salt and vinegar chips (Frito-Lay North America, Inc. Plano, Texas, USA). The outer shells for the bell pepper and rolling pin were both manually fabricated by dip molding the original objects in silicone rubber (Elastosil, WackerChemie AG, Munich, Germany). Each shell included seven coats of silicone rubber (total thickness approximately 5 mm) and powdered pigments were mixed into the final layer. For the rolling pin, a cotton sheet was wrapped around the rolling pin during curing to create a matte and rough texture. The bell pepper shell was filled with plastic pellets, and the rolling pin shell was filled with plastic filaments and table-grade salt. Each shell had a hole which enabled a connection to pneumatic tubing. The bag of chips was ready-made (i.e., store-bought and not altered), except for a small hole cut in the corner to allow a tube for vacuum and compressed air. This station also included a red and white checkered picnic tablecloth, and the side panels were painted to resemble blue sky. The **biomorphic station** was made of two objects: a long, flexible pink tube with soft spikes along the length of the tube on one side, and a flexible panel that spanned the length of the station. The pink tube was created by dip molding a 180-cm-long 5-cm-diameter PVC tube in silicone rubber (Elastosil, WackerChemie AG, Munich, Germany). The tube was rested on one side while curing, creating the spikes as excess rubber dripped off. A total of four coats of rubber were applied, and pigment was added to the final layer. The resulting rubber tube was sealed on one side and filled with wool and thread. The other side was attached to the pneumatic tubing. The flexible panel consisted of a rectangular sheet (50 cm by 245 cm) of insulation foam (Multi-Purpose Fiberglas Insulation, Owens Corning, Toledo, Ohio) encased in a thin thermoplastic urethane film. Once heat-sealed airtight and connected to pneumatic tubing on two corners, the panel was covered completely on one side with silicone rubber. While the rubber was still curing, additional insulation foam was laid on top and later removed, to create a fur-like surface texture. The **abstract station** contained two objects: a purple geometric form and a black strip. The purple form was fabricated using two open-face molds made of laser-cut acrylic pieces. Silicone rubber (Dragon Skin, Smooth-On, Macungie, PA) was cast in this mold, in multiple pours, each with a different shade of purple. The cast purple shell was filled with sawdust. For the black strip, 50 layers of 2.5 cm by 127 cm sheets of polyethylene film were encased in an air-tight heat-sealable fabric. The fabric was sealed along its edges using an iron. Both objects were also connected to pneumatic tubing. The pneumatic lines from each station were connected to a control board located outside of the experiment room. A miniature solenoid valve (991-003062-026, Parker Hannifin, Hollis, NH, USA) and an Arduino Uno Rev3 Board were used to alternate between supplying vacuum at 70 kPa and air at 10 kPa to each station, based on a specific cadence created for each station (see SI S2 Fig for specific cadence). The control was fully open-loop, there was no feedback mechanism that responded to participants’ actions. The transition dynamics between jammed and unjammed states were influenced not only by internal pressure changes but also influenced by the nature of interaction. For instance, actions such as massaging could accelerate the transition by facilitating air redistribution. Additionally, mechanical characterization tests were conducted for each of the objects in the jammed and unjammed state, to quantify the stiffness and elasticity changes. The experimental details and the results of these characterizations can be found in the Supporting Information (S1 Fig and S1 Table). However, perceived stiffness also depended on how and where participants applied force – for example, bending while twisting could alter the felt resistance compared to pure bending.

Each station had two cameras to record the actions of the participants from two angles and were all situated in a private room. Before entering the room, participants were given a card that told them in which order they would explore the stations. This order was randomized across all participants, and the stations were referred to as the blue, pink and yellow stations, in order to not prime the participants. Participants were instructed to spend time in each station until they heard a bell sound (which occurred in 5-minute intervals). Participants were given no formal instructions on how to engage with the stations. They were encouraged to play with the objects, and it was emphasized that there was no right or wrong way to engage with the objects. Once they had visited all three stations, they were asked to complete a short Qualtrics survey administered on a laptop in the experiment room.

Forty participants with a mean age of 32.8 (range 20–55) took part. Eighteen participants identified as female, 21 as male and one participant preferred not to say their gender. This study was approved by the Harvard Cambridge Area Institutional Review Board (IRB21–0843). Participants were recruited through advertisements (posters, email lists, and word of mouth which described the experiment as an “interactive haptic installation”) between July 20 and August 30, 2021. All participants provided written informed consent before participation.

The resulting 120 videos were hand-coded using DataVyu, an open-source software package developed for coding behaviors in video footage (Databrary Project, New York University, NY, USA). Each time a participant performed a behavior that fit in one of the four IP categories described above, it was manually marked with a start and finish timestamp in DataVyu. The coding was performed in a randomized order between all 40 participants by one of the authors. The processing resulted in 120 separate CSV files, with the start and finish times of each action reported in milliseconds.

A reliability analysis was performed to validate the IP categorization. A quarter of the total number of videos were taken, based on 10 randomly chosen participants from the total of 40. From these 30 videos, individual video clips were extracted based on the first coders DataVyu files. Specifically, each individual IP coded by the first coder was saved as an individual short clip, without a label identifying which IP category it belongs to. Another author independently coded these individual clips. A reliability score was obtained by comparing the labeling of the second coder to the original labeling by the first coder.

The Qualtrics survey results were also exported as CSV files. All the data was then imported into MATLAB for further processing.

## Results

### A. Emergent interactive procedures (IPs)

**Fig 2:**
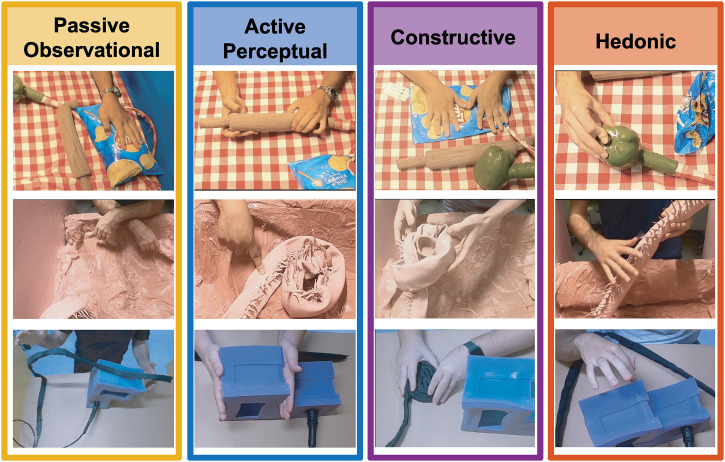
Four categories of interactive procedures (IP) emerged reliably across all stations: passive observational, active perceptual, constructive, and hedonic. The images are stills from videos recorded during experiments. See Supporting Video for more examples of the emergent behaviors observed.

As expected, participants performed distinct and reliably observable IPs that could be linked to self-determined goals. Four categories were identified: *passive observation, active perception, constructive,* and *hedonic*. These procedures have observable features indicating intent, which enable them to be reliably scored from video recordings. Transient gazes or incidental hand contacts that were not apparently directed toward a specific part or feature of an object were not considered indicators of any IP. (See Fig 2 and Supporting Video.)

**Passive Observational IPs** are performed to learn about properties of objects that can be perceived with minimal manual exploration, such as shape and coarse-scale roughness. Not only is active contact unnecessary to perceive these properties, but it might actually interfere with a perceptual goal (e.g., lifting a compliant object could change its shape boundary). Indicators of passive observation include hovering hands over an object, stepping back from an object while maintaining fixed direction of gaze, applying extended static contact to an object without any motion, or gaze-tracking along contours as indicated by head and/or eye movement.

**Active Perceptual IPs** are performed to perceive intrinsic object properties such as deformability or weight, which can only be perceived with physical intervention. These include actions such as pressing to perceive stiffness, lifting to perceive weight, and stroking or rubbing to perceive texture. Many of these actions are what have been termed exploratory procedures in prior research.

**Constructive IPs** are performed to create new shapes and arrangements with existing objects. This includes actions like stacking, coiling, folding, flattening, making knots or dents, and creating self-supporting structures (e.g., arch) [37]. The attempts for change do not necessarily have to succeed, as the procedure depends on the interaction goal. For example, a folding action is still constructive if the object unfolds due to its elastic properties, or a stacking action is still constructive even if the created stack of objects collapses.

**Hedonic IPs** are actions performed to elicit a desirable sensory experience. This includes actions such as stroking one object with another, repeatedly flicking elastic components, massaging objects for an extended period of time, or imitating purposive actions that are contextually inappropriate (e.g., wielding an unfamiliar object as one would a hammer).

When differentiating IP categories for purposes of coding, specific indicators of the primary purpose of an interaction were considered. Prior research indicates that perceptual exploration is efficient and optimized for purposes of determining object features. Actions that lacked these features might have perceptual consequences but were deferred to another category. For example, repeatedly banging an object on a surface might help gain information about its stiffness or weight but is neither efficient nor optimal and instead would be considered hedonic. Conversely, observational, perceptual, or constructive IPs might elicit pleasant sensory experiences but clearly have other goal-driven intentions.

Although this research began with a priori expectations that interaction patterns would be extended beyond the haptic exploratory procedures previously associated with explicit perceptual goals [1] to include constructive and hedonic reactions as well as passive gaze, additional categories beyond the reported four IPs were considered but ultimately rejected. More refined categories, such as two-part vs. multi-part constructions or slow vs. fast movements, were eliminated due to highly skewed likelihood across the stations, unreliable coding, or because they crossed boundaries of our principal set of IPs. Emotional categories were excluded because they tend to reflect internal states that are not directly linked to intention, and the hands do not provide salient signals of affect.

The reliability of the interactive procedure categorizations was assessed through an inter-rater agreement analysis. The two independent coders showed a raw agreement rate of 89.2% across the four main IP categories. To account for agreement occurring by chance, we also calculated Cohen’s Kappa which yielded a value κ = 0.84, with a 95% confidence interval of [0.81, 0.87], indicating almost perfect agreement. These results suggest that the coding scheme was consistently interpreted and reliably applied by both raters.

This rating matches or exceeds previous scoring of haptic exploratory procedures [1,14,38]. The start and end times of each IP were coded, allowing analysis not only of the number but also the duration and chronological sequence of IPs performed.

### B. Interactive procedure dependence on objects

**Fig 3:**
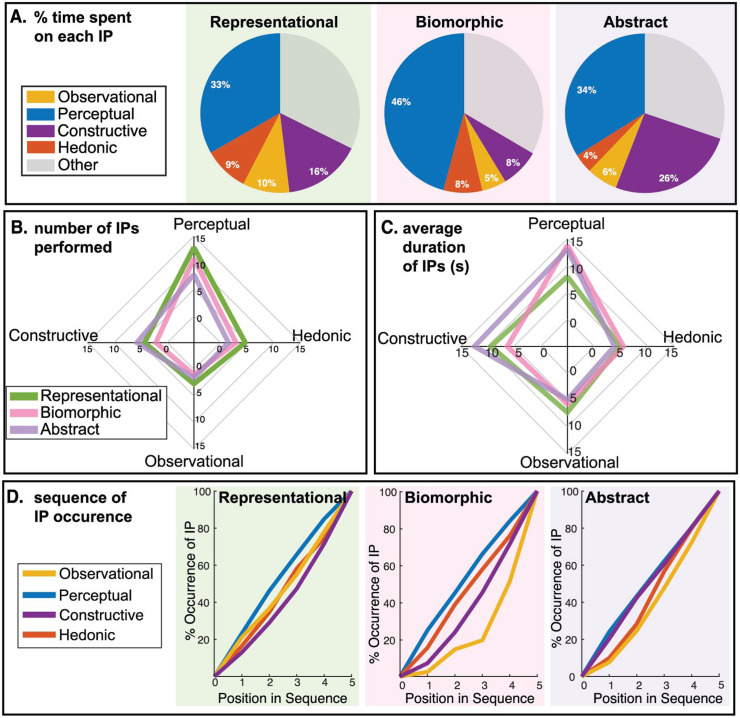
Interactive procedure patterns vary at different stations. (A) Pie charts demonstrate how participants spent their time at each station. Each pie chart sums to a total of 5 minutes. The “Other” category consists of the time participants weren’t performing any actions or were performing task maintenance (e.g., reorienting objects to prepare for next action). **(B)** Radar plot shows the number of specific IPs formed at each station. Constructive actions are clearly performed with higher frequency at the abstract station. **(C)** Radar plot shows the average duration of individual IPs (first averaged for a specific participant, then averaged across participants). **(D)** The order of emergence of different procedures types also vary between stations.

The three stations in the experimental setup were designed for a variety of affordances and relation to prior knowledge, enabling top-down and bottom-up elicitation of behavior. Analyzing how IPs were performed across the three different stations sheds light onto if and when object characteristics might influence behavior. A summary of the results separated by station and IP categories can be seen in Fig 3.

Two-way repeated measures ANOVAs were performed with station and IP category as the factors, for the three outcome measures of the interaction depicted in Figs 3A–3C: number of times an IP was performed, its average duration, and percentage time spent on a specific IP relative to total interaction time. An adjusted alpha value of 0.004 was used to determine statistical significance considering the 12 different comparisons possible with three stations and four IP types. The main effect of station was not significant for the time spent performing IPs or the average duration of an IP (p-values of 0.50 and 0.21), but station did have a significant effect on the number of IPs performed (p = 1.6*10^−9^, η²_p_ = 0.73). This results from a higher average number of IPs in the representational station (25 IPs) than the others (both 17 IPs).

The main effect of IP category was significant for all three variables: number of IPs performed, IP duration, and percentage of time spent on an IP (p-values of 1.7*10^−11^ and 1.3*10^−23^, and 4.9*10^−11^ and η² _p_ values of 0.73, 0.28, and 0.61, respectively). Across all stations, active perceptual IPs dominated in the amount of time (Fig 3A) and number of distinct IPs performed (Fig 3B), whereas the average duration showed that hedonic IPs were shorter relative to the other categories (Fig 3C). The interaction between IP type and station showed significance in two of the ANOVAs (p-values of 6.8*10^−5^ for IP duration, and 1.4*10^−9^ for percentage time spent on IP, and η² _p_ values of 0.12 and 0.2 respectively), indicating that the observed differences between IP types further vary with stations, as predicted from the hypothesis that station-specific affordances and object familiarity would affect interactions. However, the post-hoc power analysis indicates a relatively low statistical power. This suggests that while the effect is unlikely to be due to chance in this dataset, the estimate may be unstable or sample-dependent. The interactions reflect the trends that constructive IPs were more prevalent in the abstract station, whereas passive observational IPs were more prevalent in the representational station. This is demonstrated across all three outcome measures. While the links between features of each station and the interactive procedures cannot be directly determined, the nature of the specific object properties at each station suggests how it might encourage certain kinds of actions. In the abstract station the hole and the groove in the purple form are salient features that encourage constructive IPs. In fact, 53% of the constructive actions in the abstract station involved building a physical relationship between two objects, compared to 33% and 24% in the representational and abstract stations. Additionally, the abstract geometry of the object limited the amount of top-down prior knowledge possible. This therefore, made the station more suitable to construction. The greater frequency of observational IPs in the representational station may arise from the passively visible effects of jamming on the chip bag, causing shape and volume changes.

The chronological sequence of IPs at each station indicates that participants were motivated to actively interact with the displayed objects (Fig 3D). Active perceptual IPs tended to occur earlier, independent of station, whereas passive observational IPs occurred at later stages of interaction, particularly in the biomorphic and abstract stations. Constructive IPs were also observed early in the abstract station, where they were also the most frequent. The affordances for conjoining parts in this display clearly created impetus for structural creation.

### C. State & state change influence on interactive procedures

**Fig 4:**
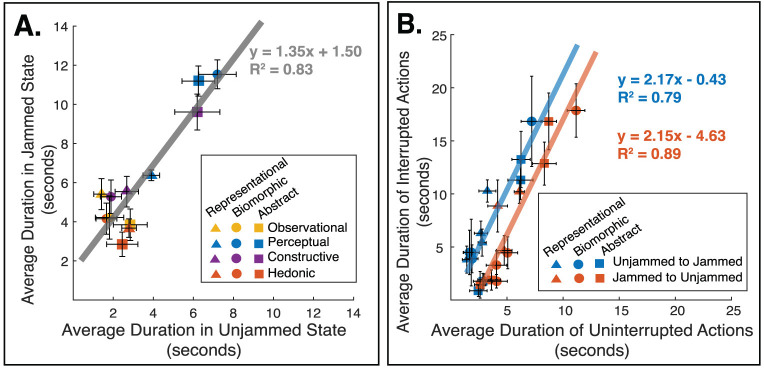
State and state changes influence interactive procedure patterns. **(A)** States influence the length of individual interactive procedures. Specifically, IPs are on average 36% longer in the jammed state, irrespective of IP type or station. (N = 40) **(B)** State change itself lengthens a procedure. Specifically, when a state change occurs while the participant is performing a procedure (i.e., the IP is interrupted by a state change), the action is twice as long compared to an equivalent uninterrupted procedure. This trend is similar regardless of the direction of change. (N = 40) (All data points are averages across all forty participants and error bars show the standard error).

As discussed in the introduction, the present research extended the concept of affordances to changes in objects’ states, with impact on their interactive potential. While the participants were interacting with each station, the objects were cycled by a jamming process between stiff (jammed) and soft (unjammed) states. The inter-state intervals were irregularly spaced to avoid rhythmic predictability (see SI S2 Fig for details). Participants’ performed IPs were separated by the two states in which they occured, to determine the effect of the state and inter-state transitions on interaction behavior. A three-way repeated measures ANOVA was performed on the average IP durations with IP category, station, and state (jammed/stiff, unjammed/soft) as factors. An adjusted alpha value of 0.002 was used to determine statistical significance considering the 24 different comparisons possible with three stations, four IP types, and two states. Our focus was on the effect of state, which was significant (p = 2.4*10^-12^, η²_p_ = 0.72). IPs were 35% longer in the jammed state compared to their counterpart in their unjammed state, independent of the station or the IP category (Fig 4A). This finding that participants have longer interactions with objects when they are rigid could stem from multiple causes including action affordances, familiarity (or conversely surprise), or aesthetic response.

Since the state changes in the stations occurred independently of ongoing actions, a state change could occur while a participant was in the middle of an interactive procedure. A further analysis separated IPs that occurred in a single state (uninterrupted by a change) from the same IPs which continued across a state change. To confirm that participants were deliberately continuing an action after the object transition, the duration after a state change was only considered in the analysis if it lasted longer than 3 seconds. This value was based on a prior study measuring the duration of a set of simple manipulation tasks as an average of 3 seconds (including the pre-contact time as well as the manipulation) [39]. The results showed that IPs interrupted by a state change were more than twice as long as their uninterrupted counterparts (Fig 4B). This trend was independent of direction of the state change (unjammed to jammed or jammed to unjammed). The up-scaling of responses after state transition suggests that a change in object affordance can be a potent driver that sustains an ongoing pattern of interaction.

### D. Subjective perception and IP action

Participants completed a survey immediately after their interactions with the full installation. A series of Likert scale questions were answered separately for each station. To assess the element of surprise at each station, responses to the following two questions were consolidated: “The objects in this station behaved in ways that surprised me,” and “The objects in this station behaved in ways that seem familiar in everyday life (reverse scored).” To assess the extent to which the station invited touch [11], responses to the two questions: “Touching the objects in this station felt good,” and “Once I touched the objects in this station, I wanted to keep touching them.” were consolidated. (The full survey results can be found in SI S3 Fig)

**Fig 5:**
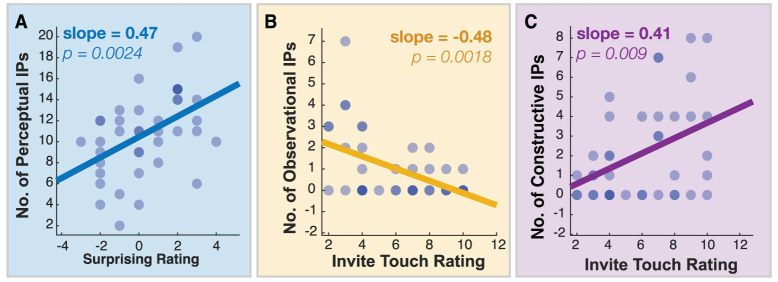
Significant linear correlations between the number of IPs performed by the participants in the biomorphic station and the two rating scales, with regression slope and significance level. (A) Perceptual IPs with Surprising rating; (B) Observational IPs with Invite Touch Rating; (C) Constructive IPs with Invite Touch Rating. (Multiple participants contributing to the same data point are shown by a darker symbol.

Linear correlations were computed to assess links between how participants rated each station and the number of IPs of a given class they performed in that station. Significant correlations were found for the biomorphic station, as shown in Fig 5. When the participants found the station’s behavior surprising, they tended to perform more perceptual IPs directed towards extracting object properties. If the participants rated the station as inviting touch, they tended to passively observe less often and perform more constructive IPs. These trends are consistent with the idea that initial responses to the environment based on expectation or affordance motivate particular patterns of interaction. Here, the linkages from surprise to active perception, and inviting touch to construction, are evident only in the relatively unstructured environment of the biomorphic display.

## Discussion

The approach of the present study was interdisciplinary, featuring a multi-station interactive art installation, which utilized current technologies from engineering and robotics research, as a vehicle for analysis according to frameworks from psychological science. Participants engaged with diverse sets of state-changing objects without specific instructions, allowing spontaneous self-directed behaviors to emerge. In this novel context, the study was able to demonstrate the emergence of systematic interaction patterns and showcase how static and dynamic affordances as well as prior knowledge and expectations modulate interactive behavior. While prior studies show how people perform specific hand gestures to achieve predetermined perceptual goals, the present study expands on this mechanism and demonstrates how individuals self-determine interaction goals based on affordances and prior experiences, and perform gestures that satisfy these goals.

In alignment with a priori expectations, the interactive procedures that emerged went beyond the classical definitions of exploratory procedures and indicated distinct underlying interaction goals beyond perceptual ones. These were identified as *observational*, *perceptual*, *constructive* and *hedonic*. The defined IP categories, which were reliably scored, reinforced and expanded on prior work demonstrating that actions are optimized to achieve specific goals. For example, to assess whether an object deforms under its own weight while resting on a surface, passive observation is appropriate, since active perceptual exploration would introduce new deforming forces. Similarly, if the goal is to combine two shapes into a new construction, an interactive procedure of careful placement is appropriate; in contrast, repeatedly banging components together may be enjoyable but does not construct a stable new form. These validated categories of interaction offer a structured foundation for designing user experiences more intentionally—for example, distinguishing between passive observation and active perception can inform the balance of visual and tactile cues.

To test if and how knowledge-driven expectations and object-specific affordances depend on the physical environment offered, the experimental setup included three distinct stations. The representational station offered expectation-driven interactions with familiar objects (chip bag, pepper, rolling pin), the biomorphic station included unfamiliar objects with organic and unformed morphologies (colors, shapes, and textures suggesting biological forms), and the abstract station included affordance-driven objects with distinct formal features (such as lines, grooves, holes). We found that the physical differences between the stations did lead to variations in the overall pattern of interactions. Specifically, participants performed more constructive IPs in the abstract station, which specifically took advantage of the affordances offered by the objects. On the other hand, participants performed more passive observational IPs in the representational station, suggesting prior-knowledge about objects enabled hypothesis-driven interactions focusing on the objects’ behavior, without intervention. In this experiment, multiple parameters varied simultaneously across stations, pointing to numerous features to explore in the design space while also limiting the ability to isolate direct causal effects. Future work could narrow the parameter space and identify key design and engineering features that elicit affordance-specific behaviors. More controlled studies – varying one parameter at a time between otherwise identical stations – can build stronger connections to prior work on affordance-based design methodologies [40,41]. Relevant parameters include resemblance to familiar objects, form factor, color, weight, texture, contextual cues, material properties, and stiffness modulation. Designing objects that deliberately violate affordance-based expectations – such as by weighing less than suggested by size and material – could additionally help clarify the role of surprise in shaping interaction.

Analysis of the time-course of exploration in the three stations provides further insights into emergent behavior. Earlier studies have investigated the time-course of exploratory procedures performed when participants were asked to classify objects they were touching without the assistance of vision. It was shown that quick actions that can provide coarse information about object properties were performed earlier [4]. In this study, participants were able to see the objects, and they were not specifically prompted to assess particular properties. However, there was still a general bias, across all stations, to perform perceptual interactive procedures early. The more manipulative and exploitative IPs, (constructive and hedonic), reliably appeared later during the interaction, after subjects had time to assess the objects’ affordances. These results also align with a prior study of spontaneous behavior in an interactive art installation, where participants were observed to first inspect and test, and then move on to play [42]. Following this early bias towards performing perceptual IPs, interaction appears to respond to the affordances of the particular station. In the post-perceptual period, hedonic exploration emerged in the biomorphic station featuring free-form shapes and macro-textures, whereas constructive interactions were most frequently initiated in the abstract station, where the objects’ structural features were salient. A more detailed study on the time-course of exploration – such as identifying which specific IPs tend to follow one another – could provide stronger connections to theories of embodied cognition, emphasizing the interdependence of perception and action [43,44].

A novel goal of this study was to investigate how spontaneous changes in an object’s state modulate haptic interactions. Transformations between the stiff and soft states were found to affect interactions in two ways: (1) Interactive procedures of all types were longer in the jammed state. Longer interactions with objects in the jammed state might arise because they are able to plastically deform and retain shape changes (like clay), allowing participants to iteratively restructure them. Another factor that might lengthen interactions is that objects in their jammed state are significantly stiffer compared to their unjammed state, requiring interactions with higher forces. (2) If a state change occurred during an interactive procedure, whether from unjammed to jammed or the reverse, that procedure was prolonged. This lengthening of interaction, which was essentially invariant over station and IP, suggests a general tendency toward adaptation to new affordances and exploitation of their possibilities. These findings build on and extend established theories of interaction. Dynamic, state-changing objects challenge static conceptions of affordances by introducing time-varying affordances [15], resulting in interaction possibilities which evolve in real time. This also has clear connections to HCI theories that highlight how affordances are not static properties, but emerge dynamically during specific interactions between users and technology [18,41].

Another avenue is research on the more specific effects of state and stiffness change, for example, how the absolute and relative magnitude of stiffness modulation promote or constrain user responses to it or how the timing and duration of these changes elicit particular behaviors [45–47]. Jamming is well-suited for this type of application, as it allows the design of specific stiffness ranges across objects with diverse geometries, scales, and deformation modes, while also enabling easy modulation of the time course of stiffness change [28]. In the present experimental setup, different stations had different cadences (see Supporting Information S2 Fig), and it was observed that the average duration of actions were shorter in the station which had shorter cadences. Participants also commented on the cadences of stiffness alteration in their open-ended survey responses, supporting the hypothesis that timing can have a salient influence on behavior. Future studies could investigate how the timing and rhythm of state changes affect action duration – an especially relevant question for HCI contexts where implicitly guiding action timing could enhance the user experience. This additionally opens up the possibility of responsive control. In the present study, state changes were governed by open-loop control; the behavior of the objects did not respond to participant actions. Future work could explore closed-loop control strategies, where specific interactive procedure features performed by participants trigger corresponding state changes, enabling more dynamic and adaptive interactions.

While the role of expectancy and affordance was emphasized in this study, other potential influences that might drive interaction are internal states like surprise or intrinsic motivation to touch. Evidence for these mediating pathways influencing IPs was found in the subjective ratings of surprise and inviting touch, which were elicited for each of the installed stations. Both measures were correlated with interactive behaviors, but only in the biomorphic station, which was the relatively unformed and unfamiliar environment. Specifically, surprising behavior increased active perceptual interactions while the invitation and reinforcement of touch increased structural interactions. This finding suggests that, in the absence of strong and clear external bottom-up or top-down directives from the physical environment, interactions are generated by intervening cognitive or emotional responses. Supporting this, a wide range of gestures were observed across the four IP categories, with notable variation between individuals. While some gestures were common across most participants (e.g., compressing with multiple fingers), idiosyncratic gestures were also observed (e.g., poking with the fifth digit). The bases for these preferences in terms of prior experience with art or technology, as well as personality variables like trait extraversion, would be of interest for further research. This approach, which looks deeper into individual differences, would also establish direct links to theories of affordance in HCI which highlight the socio-cultural aspects of interaction [48].

The present study also has applied implications. The interactive procedure framework developed is particularly valuable for designing interaction protocols for safe, fluent, intuitive, and rich human-machine collaboration [49–55]. The present experimental setup shares many characteristics with HCI systems designed to be immersive, exploratory and playful. In various contexts, individuals engage with hybrid virtual/physical environments – incorporating technologies such as AR, VR, and haptic feedback – often without well-defined goals. This trend is reflected in the growing use of virtual tours and walkthroughs across industries such as real estate, cultural heritage, art museums, galleries and retail spaces, as well as computer games where open-ended exploration is a core user experience design principle. Multi-modal interfaces are increasingly integrated into these environments; for example, incorporating haptic interaction when viewing sculptures has been shown to increase the total time spent with the artwork [56]. Similarly, our findings demonstrate that state change can lengthen interactive procedures, suggesting that embedding state-changing features into interactive installations may further enrich engagement by extending interaction time, and allowing for participants to perceive more object features.

Our work also provides an additional novel connection to existing human-robot and human-machine interaction studies, with the use of objects that were not animal-like or humanoid [54,57,58] and do not directly suggest intelligence, intentionality, or communication capabilities. This approach is especially salient as robotic systems increasingly integrate embodied intelligence through smart and responsive materials. As the programmability of object affordances continues to advance, designing interactions through material and morphological design – rather than relying on anthropomorphic cues – offers a promising direction for future work.

## Conclusion

Physical interactions with objects are characterized by a seamless integration of haptic and visual perception with movement. The behavioral patterns and neurophysiological mechanisms of this coupling have been identified by prior research. Most studies, however, set explicit perceptual goals and constrain object sets. An open question has been whether objects intrinsically motivate patterns of exploration and exploitation. By employing novel objects within an interactive art installation, and introducing autonomous changes in form and mechanical properties, this study identified four categories of interactive procedures, representing distinct underlying goals, namely: passive observational, active perceptual, hedonic, and constructive. The results demonstrate how patterns of interaction are influenced by affordances and prior knowledge, and how temporal changes in objects’ mechanical state further moderate behavior by prompting continuous adaptation. While the study context enabled rich and naturalistic interaction, the findings may be influenced by the specific material, spatial, and aesthetic properties of the installation, as well as by the multidimensional nature of the objects’ properties (e.g., stiffness, shape, state change). Future work should assess the generalizability of these findings across diverse settings and employ controlled experiments that isolate individual parameters to disentangle the specific effects of mechanical and morphological variation. This work lays the foundations for understanding our interaction with active and programmable matter.

## Supporting information

S1 Fig Mechanical characterization experiments. These were conducted for each of the objects both in the jammed and unjammed states, in order to quantitatively describe the change in mechanical properties, namely: stiffness and plasticity. A representative force deflection curve from mechanical characterization tests for the objects is shown here. (The lighter curves show multiple trials from the same experiment.) Stiffness was calculated by finding the average initial slope of the curves. The elastoplastic response was characterized by calculating the average spring back deflection. The results from all objects can be seen in S1 Table.
(PDF)

S2 Fig The jamming/unjamming or stiffening/softening cadences for each station. These were determined based on the total volume of the objects in the station, and the rise time of the different objects.
(PDF)

S3 Fig Survey results demonstrating subjective perception of the three stations in the experiment. The bars are the means of the responses which were on a five-level scale from strongly disagree to strongly agree. The error bars show the standard errors.
(PDF)

S1 Table Relevant mechanical properties of the objects. The properties were measured by obtaining force-deformation curves using a materials testing device (Instron 5566, Illinois Tool Works, Norwood, MA, USA). Each object was tested in the fully jammed and fully unjammed state, three trials for each condition, under possible configurations which mimic haptic interactions as closely as possible: indentation on different parts of the object and bending. The jammed to unjammed stiffness ratio, the absolute stiffness values in the two states, and the percent increase in hysteresis upon jamming are reported in the table. When possible or relevant, indentation tests were performed on multiple spots on the object (top and side). For the bending tests, a three-point bending fixture was used, and the tests were performed with a support distance (SD) as reported. These tests give a general quantitative sense of the dynamic mechanical properties of the objects, but it is important to note that people applied much more complex loads to the objects than what was applied here.
(PDF)

S2 Table Station order randomization summary. Participant distribution across the six possible station visitation orders (Representational, Biomorphic, Abstract), confirming randomization was implemented across conditions.
(PDF)

S1 Movie Examples of interactive procedures. Example video clips from the experimental recordings are shown. Some videos were cropped solely to exclude participants’ faces when visible, in order to prevent identification; no other editing was performed.
(MP4)

S1 Data Datavyu output. Manually coded start and end timestamps for each behavior category, as coded by the main scorer. The xlsx file includes the data that was output from the DataVyu software. The 120 separate files for each video are merged into one spreadsheet, each as an individual sheet (all three stations for all 40 subjects).
(XLSX)

S2 Data Reliability dataset. Data for the inter-rater reliability analysis. Each line in the table is an individual interactive procedure performed. Each of these procedures were labeled separately by two independent coders, which are shown as two separate columns.
(XLSX)

S3 Data Survey results. Raw output from Qualtrics survey conducted with all participants.
(CSV)

S1 Code Processing Code. MATLAB script used to perform statistical analyses and generate the plots presented in the manuscript.
(M)
