# Supplementary figures and images for: Emergent patterns of interaction with dynamic objects
Source: PLoS One. 2025 Sep 18;20(9):e0331844. doi: 10.1371/journal.pone.0331844 (PMC12445476; doi:10.1371/journal.pone.0331844)

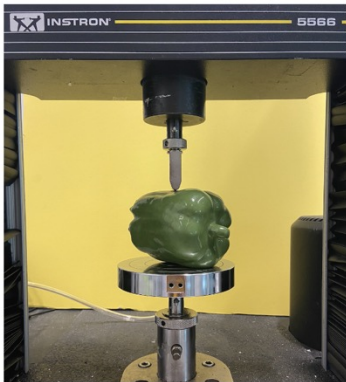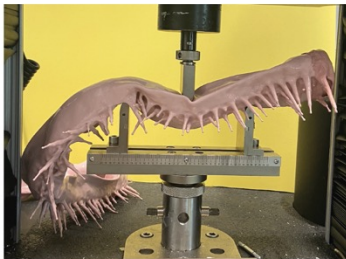

## Representative Force-Deformation Curve (from Pepper)

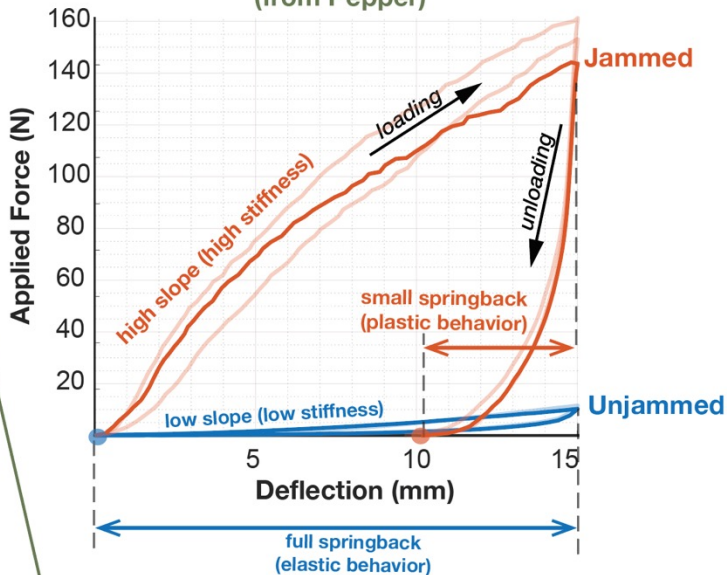

Supplement: S1 Fig — A representative force deflection curve from mechanical characterization tests for the objects is shown here. (The lighter curves show multiple trials from the same experiment.) Stiffness was calculated by finding the average initial slope of the curves. The elastoplastic response was characterized by calculating the average spring back deflection. The results from all objects can be seen in S1 Table. (PDF) [file pone.0331844.s001.pdf]

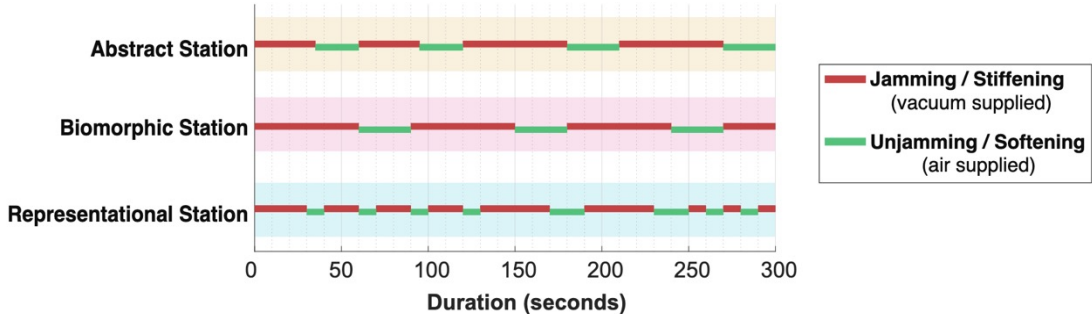

Supplement: S2 Fig — These were determined based on the total volume of the objects in the station, and the rise time of the different objects. (PDF) [file pone.0331844.s002.pdf]

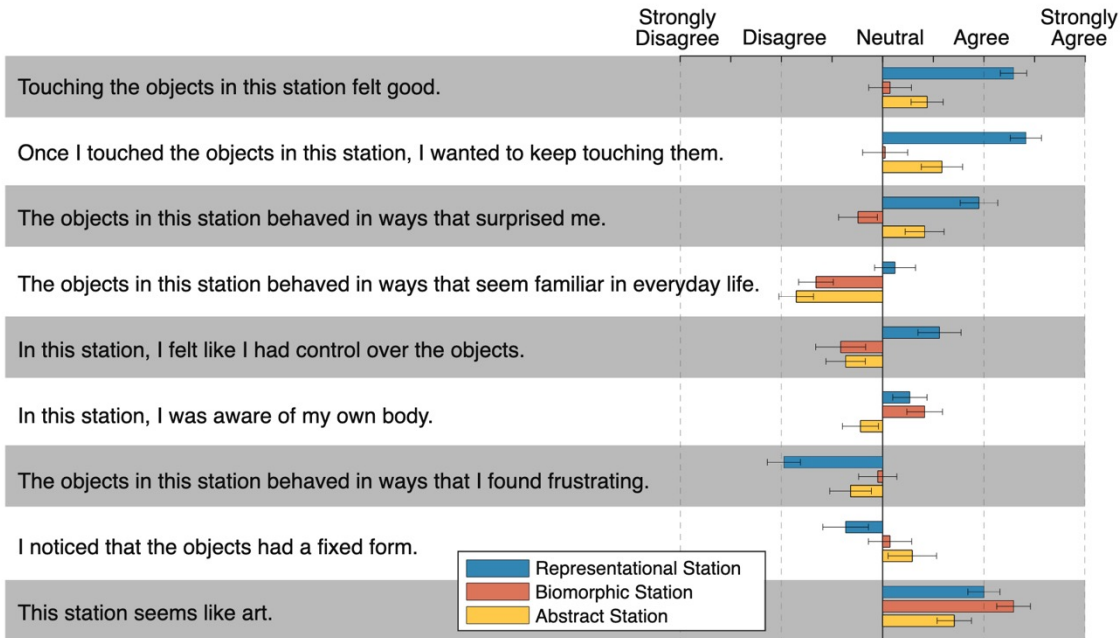

Supplement: S3 Fig — The bars are the means of the responses which were on a five-level scale from strongly disagree to strongly agree. The error bars show the standard errors. (PDF) [file pone.0331844.s003.pdf]
