# Supplementary material for: Emergent patterns of interaction with dynamic objects
Source: PLoS One. 2025 Sep 18;20(9):e0331844. doi: 10.1371/journal.pone.0331844 (PMC12445476; doi:10.1371/journal.pone.0331844)
Supplement: S1 Table — The properties were measured by obtaining force-deformation curves using a materials testing device (Instron 5566, Illinois Tool Works, Norwood, MA, USA). Each object was tested in the fully jammed and fully unjammed state, three trials for each condition, under possible configurations which mimic haptic interactions as closely as possible: indentation on different parts of the object and bending. The jammed to unjammed stiffness ratio, the absolute stiffness values in the two states, and the percent increase in hysteresis upon jamming are reported in the table. When possible or relevant, indentation tests were performed on multiple spots on the object (top and side). For the bending tests, a three-point bending fixture was used, and the tests were performed with a support distance (SD) as reported. These tests give a general quantitative sense of the dynamic mechanical properties of the objects, but it is important to note that people applied much more complex loads to the objects than what was applied here. (PDF) [file pone.0331844.s004.pdf]

Table S1. Relevant mechanical properties of the objects were measured by obtaining force-deformation curves using a materials testing device (Instron 5566, Illinois Tool Works, Norwood, MA, USA). Each object was tested in the fully jammed and fully unjammed state, three trials for each condition, under possible configurations which mimic haptic interactions as closely as possible: indentation on different parts of the object and bending. The jammed to unjammed stiffness ratio, the absolute stiffness values in the two states, and the percent increase in hysteresis upon jamming are reported in the table. When possible or relevant, indentation tests were performed on multiple spots on the object (top and side). For the bending tests, a three-point bending fixture was used, and the tests were performed with a support distance (SD) as reported. These tests give a general quantitative sense of the dynamic mechanical properties of the objects, but it is important to note that people applied much more complex loads to the objects than what was applied here.

Object	Test Configuration	Stiffness Ratio	Jammed Stiffness [N/mm] (SE)	Unjammed Stiffness [N/mm] (SE)	Jammed Springback [mm] (SE)	Unjammed Springback [mm] (SE)
Chips	Indentation Test (side)	202.7	9.65 (4.34)	0.048 (0.006)	2.31 (0.17)	2.12 (0.11)
Chips	Bending Test (10 cm SD)	4.1	0.22 (0.04)	0.054 (0.027)	4.68 (1.45)	14.68 (1.35)
Pepper	Indentation Test (side)	33.2	13.38 (0.66)	0.403 (0.034)	5.07 (0.16)	13.6 (0.38)
Pepper	Indentation Test (top)	15.9	8.27 (1.08)	0.522 (0.070)	5.55 (0.58)	13.86 (0.31)
Rolling pin	Indentation Test (side)	15.1	20.23 (1.55)	1.340 (0.362)	7.08 (0.60)	13.15 (0.10)
Rolling pin	Bending Test (15cm SD)	3.9	3.43 (0.05)	0.874 (0.025)	26.09 (0.68)	37.46 (0.48)
Pink Tube	Indentation Test (side)	10.6	6.42 (1.17)	0.605 (0.013)	8.10 (1.24)	12.57 (0.22)
Pink Tube	Bending Test (15cm SD)	2.9	0.92 (0.07)	0.321 (0.008)	23.16 (1.59)	38.79 (0.48)
Pink Panel	Indentation Test (top)	307.8	21.74 (1.23)	0.071 (0.006)	4.51 (0.31)	12.46 (0.40)
Black Strip	Bending Test (15cm SD)	3.7	0.36 (0.04)	0.097 (0.001)	26.46 (1.75)	38.12 (0.56)
Purple Form	Indentation Test (side)	2.3	7.55 (2.04)	3.280 (0.347)	10.14 (0.20)	15.01 (0.0001)
Purple Form	Indentation Test (top)	1.6	4.10 (0.24)	2.609 (0.037)	12.45 (0.30)	14.90 (0.11)
