# Supplementary material for: Emergent patterns of interaction with dynamic objects
Source: PLoS One. 2025 Sep 18;20(9):e0331844. doi: 10.1371/journal.pone.0331844 (PMC12445476; doi:10.1371/journal.pone.0331844)
Supplement: S2 Table — Participant distribution across the six possible station visitation orders (Representational, Biomorphic, Abstract), confirming randomization was implemented across conditions. (PDF) [file pone.0331844.s005.pdf]

S2 Table. Station order randomization summary. Participant distribution across the six possible station visitation orders (Representational, Biomorphic, Abstract), confirming randomization was implemented across conditions.

	**First**	**Second**	**Third**	**Occurrence**
**Order 1**	Representational	Abstract	Biomorphic	7
**Order 2**	Representational	Biomorphic	Abstract	5
**Order 3**	Abstract	Biomorphic	Representational	6
**Order 4**	Abstract	Representational	Biomorphic	8
**Order 5**	Biomorphic	Representational	Abstract	7
**Order 6**	Biomorphic	Abstract	Representational	7
